# Microbial Decolorization of Triazo Dye, Direct Blue 71: An Optimization Approach Using Response Surface Methodology (RSM) and Artificial Neural Network (ANN)

**DOI:** 10.1155/2020/2734135

**Published:** 2020-02-18

**Authors:** Khairunnisa' Mohd Zin, Mohd Izuan Effendi Halmi, Siti Salwa Abd Gani, Uswatun Hasanah Zaidan, A. Wahid Samsuri, Mohd Yunus Abd Shukor

**Affiliations:** ^1^Department of Land Management, Faculty of Agriculture, University Putra Malaysia, Serdang 43400, Selangor, Malaysia; ^2^Department of Agricultural Technology, Faculty of Agriculture, University Putra Malaysia, Serdang 43400, Selangor, Malaysia; ^3^Department of Biochemistry, Faculty of Biotechnology and Biomolecular Sciences, University Putra Malaysia, Serdang 43400, Selangor, Malaysia

## Abstract

The release of wastewater from textile dyeing industrial sectors is a huge concern with regard to pollution as the treatment of these waters is truly a challenging process. Hence, this study investigates the triazo bond Direct Blue 71 (DB71) dye decolorization and degradation dye by a mixed bacterial culture in the deficiency source of carbon and nitrogen. The metagenomics analysis found that the microbial community consists of a major bacterial group of *Acinetobacter* (30%), *Comamonas* (11%), *Aeromonadaceae* (10%), *Pseudomonas* (10%), *Flavobacterium* (8%), Porphyromonadaceae (6%), and Enterobacteriaceae (4%). The richest phylum includes Proteobacteria (78.61%), followed by Bacteroidetes (14.48%) and Firmicutes (3.08%). The decolorization process optimization was effectively done by using response surface methodology (RSM) and artificial neural network (ANN). The experimental variables of dye concentration, yeast extract, and pH show a significant effect on DB71 dye decolorization percentage. Over a comparative scale, the ANN model has higher prediction and accuracy in the fitness compared to the RSM model proven by approximated *R*^2^ and AAD values. The results acquired signify an efficient decolorization of DB71 dye by a mixed bacterial culture.

## 1. Introduction

Utilization of dye by major textile industries in their production delivers a huge volume of dye effluent, and this makes up about two-thirds of the total amount of dye production. The challenges in taking care of the textile effluent properly are greater structure variability, excessive concentration of color, and 10% being lost in coloration procedure as well as dyes will be instantly released into the aqueous effluent about 2% [1]. The causes of turbidity, an awful image, and bad smell of water are due to the colloidal matter found besides colors and oily impurities in the dye as the photosynthesis process is disrupted due to the penetration of sunlight being blocked [2]. The most severe effect of textile waste towards marine creatures causes a lack of dissolved oxygen in the water. Eventually, the self-purification process of water is stopped [3].

Additionally, decreased soil efficiency can be seen once these effluents pass in the fields and clog the soil pores. Solidified soil causes the penetration of roots to be held back. The sewerage tubes become rusted and encrusted due to the wastewater which runs inside the drains causing the changes in the grade of drinking water in hand pumps rendering it not fit for human uptake [3].

Incorporation of significant amounts of water and chemicals in the staining operations of the textile industry leads to environmental pollution and the formation of a complex, toxic, and recalcitrant residual [4]. The textile effluent needs high chemical oxygen demand and coloration with the occurrence of dyes, pigments, and additional chemicals which make the effluent require particular handling [5]. Innovative methods were explored to attenuate the environmental harm that may induce the removal of textile dyes within industrial effluents. Azo dye, made up of one or more -N=N- double bond, amounts to 60–70% of dye production [6, 7]. Physical and chemical methods including flocculation, adsorption, and photochemical oxidation are great options for the decolorization of printing and dyeing wastewater (PDW).

Several physical and chemical methods have been applied to treat Direct Blue 71 dye, including Fenton's oxidation [8], ozonation [9], ultrasound [10], and adsorption [11]. The release of secondary pollutants and large operating costs have become the main drawbacks of this method. The biological practice offers the aspects of cost-effective and simple operations in contrast to physical and chemical methods and is currently widespread in the dye treatment method [12]. Microorganisms are being intensively studied regardless of physical and chemical options available [13]. Several works with the applied of fungi and bacteria have already been designed to develop bioprocesses meant for treating textile effluents.

At the moment, the majority of isolated bacteria require anaerobic growing conditions to degrade azo dyes [14]. Nonetheless, the functional group associated with the azo dye makes up a complex composition and influences the capabilities of the bacteria. Therefore, choosing the best azo dye decolorizing bacteria is critical. Often, the deposition of toxic, mutagenic, and carcinogenic aromatic amines is coupled with decolorization of the azo dyes which are tolerant to degradation in anoxic conditions and disrupt the food chain as it accumulates [15, 16]. Thus, a key for the complete removal of azo dye from the environment is the azo dye full degradation besides only the elimination of color [17].

Presently, both response surface methodology (RSM) and artificial neural network (ANN) tools are used for the optimization and modeling of environmental research. Relationship determination among experimental variables and responses use response surface methodology (RSM). Besides that, this great technique is able to depict the main and interaction effects. Particular experimental design mixtures are used in RSM to build mathematical models with linear, quadratic, and interaction terms by a provided number of elements and response factors to get optimum operation [18]. Azila et al. [19] and Amini et al. [20] have reported studies in environmental issues in applying the RSM technique.

One of the best tools for modeling nonlinear and complex conditions is undoubtedly artificial neural networks (ANNs) out of various multivariate statistical techniques [21]. It is a strong modeling tool because of its flexibility to extend and learn the response of all nonlinear and complex processes. ANN can be effectively used in the modeling of many operations as applied by Prakash et al. [22] and Yetilmezsoy and Demirel [23]. A major cut in the number of experiments, as well as the information on singular or combination effects related to the independent variables, is possible by using multivariate statistical methods [20]. This leads to the optimization and growth of the operating system, noticeably lowering the expense of trials. The response surface methodology (RSM) and artificial neural network (ANN) are the most frequently utilized methods in research on dye decolorization literature. These two are strong data modeling tools concerning independent variables and responses of the system to gain and depict complex nonlinear interactions.

Different kinds of optimization for the handling of environmental pollution have implemented RSM and ANN techniques due to its utmost precision [24, 25]. Prediction models received from RSM and ANN have effectively optimized the lead removal from industrial sludge leachate [26], and Singh [27] used the ANN practice for methyl red decolorization in 24 hours by optimizing the design parameters of a bacterial isolate, ITBHU01.

Abd El–Rahim et al. [28] worked on optimization of metal ion concentration, pH, amount of adsorbent, and temperature using RSM and ANN, which is designed to optimize the chromium (VI) abatement by working with cyanobacteria. Similarly, Astray [29] worked on optimizing the capabilities of cyanobacteria in optimum condition predicted by RSM and ANN to degrade and decolorize distillery spent wash. Furthermore, RSM and ANN approaches were also tremendously employed in diverse fields, for example, a simulation model of ventilated room with thermal effects [30], predicting the suitability of sugar beet pulp to make oligosaccharides [31], optimizing the copper removal from synthetic wastewater by using electrocoagulation system [32], modeling the quality parameters of spray-dried pomegranate juice [33], predicting cold water temperature in a forced draft cooling tower [34], and creating a stable oil-in-water for emulsification process as well as decreasing the number of trials, time, and cost [35]. Comparison between RSM and ANN techniques was also done by Ravikumar [36] and Sen [37] for their prediction and optimization abilities.

In former studies, the mixed bacterial cultures were grown in rich media supplemented with a carbon source in anaerobic conditions to get the highest decolorization activity [38]. Yet, these complex laboratory substrates would not be suited for in situ application. It was discovered that ANN has a higher prediction ability with minimum error [36] and higher accuracy compared to the RSM model [37]. Even so, a method for mixed bacterial decolorization of Direct Blue 71 dye by using both RSM and ANN techniques has not been discovered yet. Hence, the main motivation behind this study is to optimize the bioremediation of DB71 dye decolorization by using both RSM and ANN techniques despite the absence of carbon and nitrogen source for the decolorization process.

## 2. Materials and Methods

### 2.1. Dyes, Chemicals, and Culture Media

Direct Blue 71 dye (C.I. 34140; dye content, 50%) used in this study was purchased from Sigma-Aldrich Chemical Co., USA. 10 g of dye powder was added to 1 L of deionized water for the stock solution of dye. Only analytical grade chemicals or reagents were used in this study. The isolation of dye-degrading mixed bacterial culture used minimal salt medium (MSM) (g/L: (NH_4_)_2_SO_4_, 0.4; KH_2_PO_4_, 0.2; K_2_HPO_4_, 0.4; NaCl, 0.1; Na_2_M_0_O_4_, 0.01; MgSO_4_.7H_2_O, 0.1; MnSO_4_.H_2_O, 0.01; Fe_2_(SO_4_)_3_.H_2_O, 0.01; and yeast extract, 1), whereas glucose (1% w/v) was used for the test of carbon source.

### 2.2. Soil Sampling

Samples were collected randomly from polluted and nonpolluted water, soils, and sludge sites in Malaysia. Water and soil samplings were collected in March 2018 from a depth of 8–10 cm from the water, soil, and sludge surfaces. The samples were stored in 50 mL centrifuge tubes and capped on during the transfer from the site to the laboratory. All samples were then stored at −20°C. The location of the samples was recorded based on the map coordinates provided by a global positioning system (GPS) locator.

### 2.3. Isolation of Direct Blue 71 Dye-Degrading Mixed Culture

The soil or sludge (1 g weight) or water (1 mL) samples were mixed in 50 mL minimal salt medium (MSM) under three different mediums in which MSM was added with glucose as a carbon source or without glucose and ammonium sulfate or MSM only with all the mediums supplemented with 50 mg/L Direct Blue 71 dye. All three mediums were exposed at room temperature in two different conditions which were incubated on a rotary shaker (150 rpm) and also a static condition for four days. All the different conditions were necessary to find out the best conditions that fit with the mixed bacterial culture. Then, 1% of the culture was transferred into 20 ml of the fresh medium of the same conditions as before in a universal bottle until decolorization was observed. After 7 times subculturing, 1 ml cultures were taken out aseptically and underwent serial dilutions. Then, 50 *μ*l of the diluted aliquots was spread on the Direct Blue 71 dye agar in Petri dishes based on the condition that the mixed cultures preferred referring to the addition of glucose or MSM only or without glucose and ammonium sulfate. The plates were incubated for one to three days until the bacterial colonies were visible.

### 2.4. Screening of Direct Blue 71 Dye Mixed Culture

MSM of 50 mg/L Direct Blue 71 dye was used to grow the isolate with or without glucose as a carbon source or without both glucose and ammonium sulfate and then exposed to two conditions which were static and shaken conditions for at least 4 days to screen for the highest degradation of Direct Blue 71 dye. After 4 days, 1 ml aliquots of culture fluid were pipetted out and centrifuged. Suspended particles and cells were eliminated from withdrawn samples by centrifuge (12,000 rpm) for 10 minutes to avoid absorbance readings error. The decrement in absorbance was measured from the supernatants obtained after centrifugation with uninoculated dye serving as a control relative to the dye relevant wavelength. UV-visible double-beam spectrophotometer at 587 nm wavelength was used to monitor the absorbance of the original and treated samples, and the blank was the uninoculated medium without the dye. All tests were performed in triplicate and calculation involved the average values obtained from the entire test. The decolorization percentage of DB71 was done as follows:

(1) $$\begin{array}{c} \text{decolorization}\left(\%\right)=\left[\frac{\left({\text{Ab}}_{0}-{\text{Ab}}_{1}\right)}{{\text{Ab}}_{0}}\right]\times 100, \end{array}$$

where Ab_0_ is the dye solution's initial absorbance and Ab_1_ is the dye solution's final absorbance after decolorization. Next, testing with different dye concentrations ranging from 50 ppm to 150 ppm was done for secondary screening. Isolates with the highest percentage of decolorization, constant degrading of the dye for all subculturing, can degrade high concentration of dye, and possessed special characteristics were chosen from the isolates that required neither glucose nor ammonium sulfate as their carbon and nitrogen source to live and degrade the Direct Blue 71 dye.

### 2.5. Metagenomics Analysis

Metagenomics of microorganisms applies towards discovering collections of genomes out of a mixed population of microbes in non-culture-driven methodology [39]. Studies regarding variations in bacterial and viral communities coming from diverse ecosystems were a success through genomic studies in which total DNA extraction for samples of selected mixed bacterial culture was prepared to amplify the microbial sequences [40]. The extracted DNA can then be analyzed for metagenomics to determine the microbial communities in the sample that is accountable to degrade Direct Blue 71 dye. Metagenomics analysis of the mixed bacterial culture was carried out by Apical Scientific Sdn. Bhd.

### 2.6. Optimization of Decolorization Using Response Surface Methodology (RSM)

A practical design by RSM was proposed for modeling and analysing tasks holding different parameters associated with mathematical and statistical methods as well as to optimize a method with a practical utilization of the resource elements [41]. It is also designed for projecting the functional relationship involving experimental parameters and the response [42]. The optimum levels of three significant parameters, including dye concentration, yeast extract, and pH, were identified through the RSM approach besides determining the relationship concerning the input parameters and the response functions. Design-Expert 6.0.8 was used to evaluate the acquired outcomes. Model evaluation was done by comparing the RSM predicted values with the experimental values [43]. Evaluation of the experimental results was done through a number of regressions, and the *F* test was calculated to obtain the significance of the regression value [44]. Values of *R*^2^ (coefficient of determination) and adjusted *R*^2^ must be near to 1.0 for a good relationship for the model which concerns both the predicted and experimental values [45]. The regression coefficients obtained from the regression model are helpful for response surface plotting [46].

### 2.7. Optimization of the Significant Parameter Using Box–Behnken Design

The Box–Behnken design was used to optimize the decolorization of Direct Blue 71 dye in RSM. The process includes the combination of treatment at the midpoints and center which are unbiased, rotatable, or almost rotatable quadratic design [47]. Contrary to the other RSM models, this design requires less experimental tests and a shorter period [48]. Therefore, it gives an extra economical approach. Next, statistical analysis of the acquired outcomes was done by using Design-Expert 6.0.10, Stat-Ease, Inc., Minneapolis, USA [49]. Dye concentration (A), yeast extract (B), and pH (C) were the independent variables, and the design generated 17 total experiments in which the percentage of decolorization is the response. An ideal model ought to have insignificant lack of fit and a significant model. The significant terms were determined for every response. With respect to three parameters (*n* = 3), as shown in Table 1, and limits which were an upper limit and a lower limit, the total number of the test was 17. The response was determined as the percentage of dye decolorization. In order to minimize the variability which was uncontrollable, as a result from the observed responses, the experiment was done in a randomized method [50]. The model was evaluated from statistical significance obtained from analysis of variance (ANOVA) and response surface plot, and regression equation was analyzed to acquire the optimal values.

### 2.8. Optimization of Direct Blue 71 Decolorization Using Artificial Neural Network (ANN)

Several learning algorithms were used to train the networks by using NeuralPower version 2.5. Only one hidden layer was applied to identify the optimal network topology, and several networks were established by constant identification of the transfer function and the number of neurons in this layer. The network was trained until the network root of mean square error (RMSE) was lower than zero; the average determination coefficient (DC) and average correlation coefficient (*R*) were 1. Training started by random values, and over the training process, it was adjusted to reduce network error [51].  Table 2 shows two sets which were unbold training dataset and bold testing dataset, and the validating set was the experimental and predicted values of ANN at optimum conditions.

### 2.9. Residual Analysis (Error Analysis)

Comparison between predicted response from the RSM and ANN models was done to assess the dependability of the estimation potential of the applied methods [52]. A model's accuracy cannot be dependent only on *R*^2^ as calculated using equation (2). Therefore, the implementation of AAD analysis was required as an immediate practice for explaining the deviations by using equation (3). *R*^2^ and AAD were calculated, respectively, by using the following equations:

(2) $$\begin{array}{c} R^{2}=1-\frac{\sum_{i=1}^{n}{\left(\text{model }{\text{prediction}}_{i}-\text{experimental }{\text{value}}_{i}\right)}^{2}}{\sum_{i=1}^{n}{\left(\text{model }{\text{prediction}}_{i}-\text{experimental }{\text{value}}_{i}\right)}^{2}}, \end{array}$$

(3) $$\begin{array}{c} \text{AAD}=\left\{\frac{\sum_{i=1}^{p}\left(\left(yi_{\exp}-yi_{\text{cal}}\right)/yi_{\exp}\right)}{p}\right\}\times 100, \end{array}$$

where *yi*_exp_ were the experimental responses and *yi*_cal_ was the measured responses, the number of experimental runs was denoted by *p*, and the number of the experimental data was denoted by *n*. Accuracy of the chosen model was confirmed through *R*^2^ and AAD value analysis in which *R*^2^ is close to 1 and a small AAD value is obtained [53, 54]. This implies that the actual behavior of the system was successfully described by the model equation with the best values of *R*^2^ and AAD values [53].

### 2.10. Determination of Optimum Point

Desirability function method was used for identifying the predicted optimum state by RSM and ANN. Each parameter requires desires and priorities to be involved in this method to develop a process for identifying desirability of the responses and the relationship between the percentage of dye decolorization on each parameter [48]. Variables of dye concentration, yeast extract, and pH were fixed at maximums at the trial level [55, 56].

## 3. Results and Discussion

### 3.1. Isolation, Screening, and Identification of Mix Culture Using Metagenomics Analysis

This study was conducted to isolate and screen a potent Direct Blue 71 dye decolorizing mix culture from Malaysian soil samples. All thirty samples that were collected were tested with three different media supplemented with DB71 dye in which the carbon source came from the glucose while the nitrogen source came from the ammonium sulfate before being put under static and shaking conditions. Mix culture from the soil from Tasik Sri Serdang (*N* = 3°00′15.8″, *E* = 101°42′48.9″) has been selected as the most promising culture that is able to degrade Direct Blue 71 dye because of its constant decolorization even after seven times subculturing and can degrade higher concentration of dye compared to the other samples. Furthermore, this chosen mix culture shows an excellent ability to survive without both carbon and nitrogen sources in static conditions, leaving yeast extract in MSM as the sole organic nitrogen source for the mixed culture. Numerous azo dye decolorization research studies were achieved in the existence of supplemental carbon and nitrogen sources. Nevertheless, the added carbon source was chosen by the cell over the dye compound which made the decolorization by supplementing carbon source turn out to be much less effective [57]. Ultimately, this study successfully isolated mix bacterial culture that effectively uses the dye as a carbon source and decolorizes the dye efficiently without external carbon and nitrogen sources.

The receiver of reduced electron carriers between oxygen and azo compounds in aerobic conditions caused competition between those two compounds, and it might be the factor for successful decolorization in static conditions. This reaction was the same as Handayani et al. [58], who published the decolorization by *Enterococcus faecalis* for Reactive Red 2 and Acid Red 27 in a batch system. Disruption in the complex form of enzyme molecules from mechanical shake was so much that deactivation arises. Mechanical frailty's trait of enzymes in shaken condition causes the increment in oxygen transfer rates and substrate mass transfer [59]. Hence, dye decolorization in the anaerobic condition is a serendipitous process as the electron acceptor would be the dye [60].

The mixed culture was adapted to high dye concentrations as they were collected from contaminated sites near the lake at Taman Tasik Sri Serdang. Similarly, Chen et al. [38] reported the isolation and screening of microorganisms from sludge samples that are able to decolorize several azo dyes. The samples were obtained from a wastewater treatment plant and lake mud. Microbial decolorization of toxic dyes relies upon the adaptability as well as the reaction of the chosen microorganisms towards dyes [28]. Oxygen curbs the azoreductase enzyme responsible for microbial decolorization of dye because of the match between electron acceptor for the oxidation of NADH which are the oxygen and azo groups [61]. Just a small quantity of oxygen is transferred, most likely on the broth surface in static incubation leading to decolorization in anaerobic conditions being carried out by the cells deposited towards the bottom of the flasks [62].

**Figure 1:**
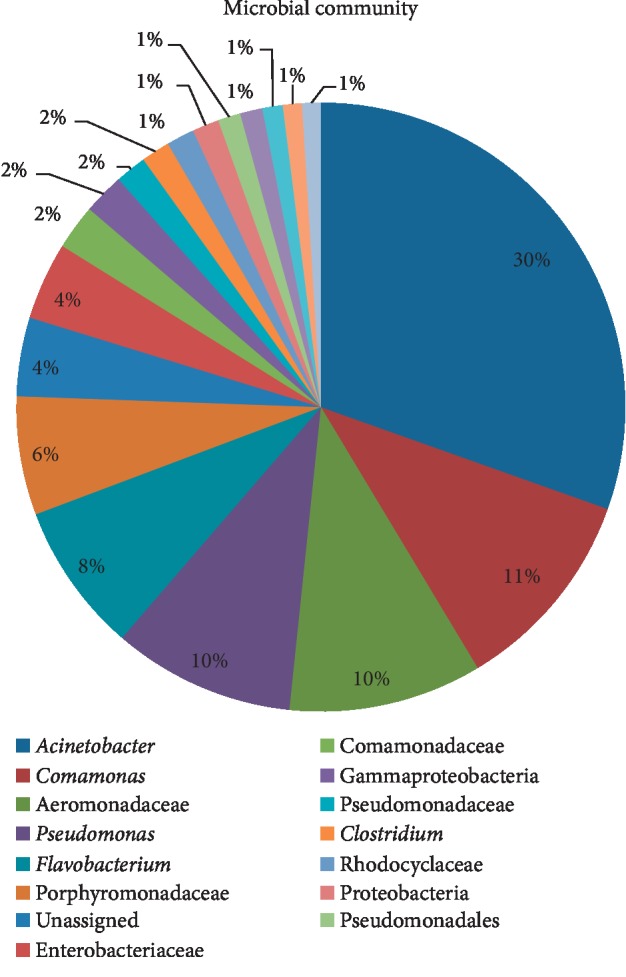
Microbial community found in the mixed bacterial culture.

Metagenomics analysis of the mixed bacterial culture shows *Acinetobacter* was the dominant bacterial group, followed by *Comamonas*, Aeromonadaceae, *Pseudomonas*, *Flavobacterium*, Porphyromonadaceae, and Enterobacteriaceae, as represented in Figure 1, respectively, 30%, 11%, 10%, 10%, 8%, 6%, and 4%. The most abundant phylum was Proteobacteria (78.61%), followed by Bacteroidetes (14.48%) and Firmicutes (3.08%). A study by Ghodake et al. [63] reveals that *Acinetobacter* was identified to decolorize 20 diverse textile dyes of various classes. Some *Acinetobacter* strains as a biocatalyst has been applied to remediate several environmental pollutants along with biotechnological uses reported by Abdel-El-Haleem [64]. The effluent-adapted strain of *Acinetobacter* has the potentiality for color removal, and strains of *Acinetobacter* and *Pseudomonas* exhibit chemical oxygen which demands removal actions [64, 65]. Besides that, the *Comamonas* strain retains amazing reusability and endurance traits in continuous decolorization processes [66]. Additionally, the genus of *Pseudomonas* including *Pseudomonas putida* and *Pseudomonas alcaligenes* has a lot of metabolic diversity, many of which were capable of metabolizing different chemical contaminants [67]. Also, it is reported that Proteobacteria was a dominant part of the mixed bacterial culture in anaerobic-baffled reactors useful to deal with industrial dye wastewater [68].

### 3.2. Optimization of Direct Blue 71 Dye Decolorization Using RSM

In this study, response surface methodology (RSM) was used to optimize the decolorization of DB71 dye. The response surface equation from Design-Expert® can be optimized for the best result considering a range of process variables. The effect between the two parameters was shown on response plots, and the relationships were shown on contour by maintaining other parameters specified at their best optimal conditions [69]. From these contour plots, the relationship of one variable to the next could be seen. Lastly, the hump on the contour plots determined the optimum condition for every variable [70].

Optimization of anaerobic mixed bacterial culture degrading DB71 dye with the absence of carbon and nitrogen source using RSM is a novel approach. Bacteria are seldom able to decolorize azo compounds in MSM lacking in nitrogen source as stated by You and Teng [71]. A lacto bacterium degradation performance is very slow when MSM-lacked nitrogen source is used compared to the rapid degradation of Reactive Black 5 obtained only in one day within a medium with external nitrogen addition [71]. However, in comparison with a previous study, the chosen mix culture for this study is extra applicable for in situ application as it does not need ammonium sulfate that acts as the nitrogen source for an efficient DB71 dye decolorization.

The combined effect of significant parameters that includes dye concentration (A), yeast extract (B), and pH (C) for decolorization of DB71 dye was studied, and these parameters were optimized to acquire the highest decolorization percentage using a Box–Behnken design with 17 experimental runs.  Table 2 shows the experimental and predicted responses by using RSM and, later, the responses were analyzed using Design-Expert 6.0, Stat-Ease Inc., Minneapolis, USA.  Table 2 shows that dye decolorization by mixed bacterial culture ranged from 54.43% to 94.9% at run 6 and run 3, respectively. The maximum dye decolorization (94.9%) was obtained at dye concentration (A, 50 ppm), yeast extract (B, 3 g/L), and pH (C, 6.75) in run 3. The minimum dye decolorization (54.43%) obtained at dye concentration (A, 150 ppm), yeast extract (B, 0.5 g/L), and pH (C, 6.75) was observed in run 6.

### 3.3. Final Equation in terms of Coded Factors

Analysis of variance (ANOVA) for the predicted RSM model is shown in Table 3 for DB71 dye decolorization. The equation for the regression model was as follows:

(4) $$\begin{array}{c} \text{decolorization}=+89.84-5.90^{*}\text{A}+13.53^{*}\text{B}+2.35^{*}\text{C}+4.89^{*}\text{AB}-1.72^{*}\text{AC}-2.44^{*}\text{BC}-1.930^{*}{\text{A}}^{2}-8.340^{*}{\text{B}}^{2}-6.49^{*}{\text{C}}^{2}. \end{array}$$

Table 3 shows a significant model for optimizing DB71 dye decolorization with low probability value (*F* < 0.0001) and *F* value of 47.15. Only 0.01% chance of error due to noise could occur to this *F* value. Model terms were significant when *p* > *F* values were less than 0.05, and model terms were not significant when values were higher than 0.10 [72]. Therefore, in this study, A, B, C, AB, BC, B^2^, and C^2^ were significant model terms. Adequate precision shows an adequate signal for the model, which was 21.101. PRESS shows the goodness of the model to predict the responses in new experimentation which was 435.07 for this model. The model fits the data as the value of 2.35 for lack-of-fit *F* test was statistically not significant. Determination coefficient (*R*^2^) was used to validate the model's goodness of fit which was 0.983 for this study, and reasonable agreement with the predicted *R*^2^ (0.8252) indicated an adequate prediction for DB71 dye decolorization. A good prediction of a model was implied by the closeness of the *R*^2^ value to 1.0 [73]. Unreliable outcomes in the prediction analysis could be prevented by having a fit model in an optimization study.

**Figure 2:**
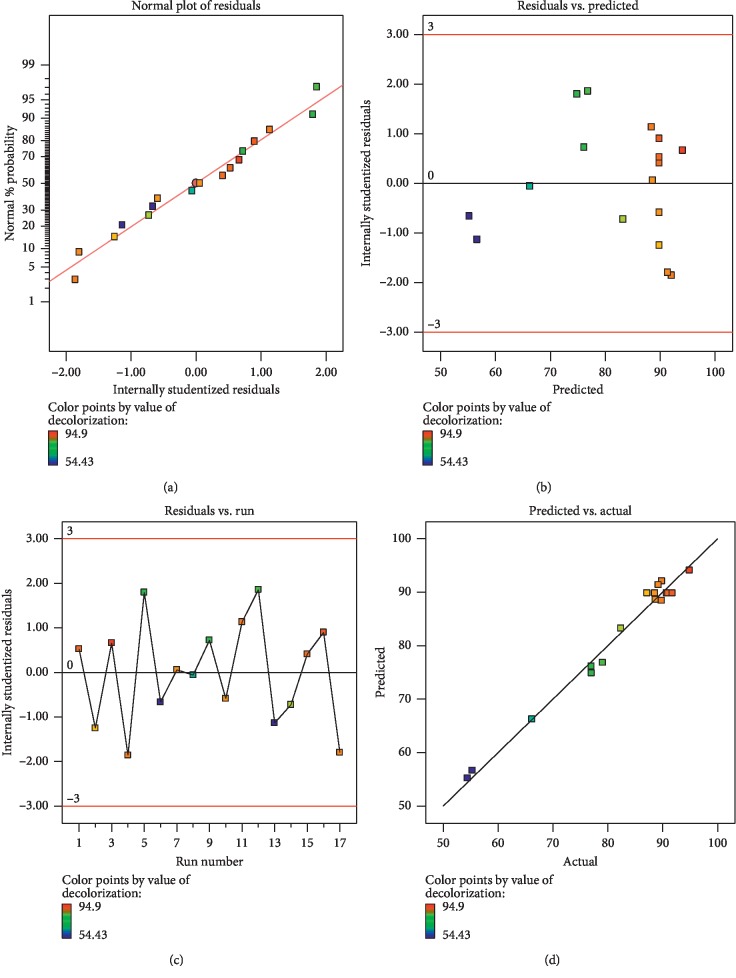
Diagnostic plots showing (a) the studentized residuals plotted against the normal probability, (b) the studentized residuals versus predicted, (c) the studentized residuals versus run, and (d) the predicted response values plotted against the actual responses.

A straight line of the studentized residuals plotted versus the normal probability from Figure 2(a) shows that the experimental data displayed a normal distribution. No data error was found in the estimation of the model as all values lie within the interval ±3.00, as shown in Figures 2(b) and 2(c). Correlation coefficients (*R*^2^ and *R*^2^ adj) of 0.98 and 0.96, correspondingly, for decolorization of DB71 dye fit one another as is displayed through a plot of actual versus predicted response values as shown in Figure 2(d). As a result, the established model was appropriate for predicting the performance of dye decolorization within investigated conditions.

### 3.4. Optimization Using Artificial Neural Network

A crucial selection of neural network topology was required for an effective treatment. Therefore, prediction of DB71 dye decolorization requires the analysis of different neural network topologies. The best four ANN models were outlined based on Table 4. One model from the list of models was chosen to lower the cost criterion of training a neural network model. The best option for the learning algorithm was batch backpropagation (BBP) for the prediction of DB71 dye decolorization (Table 4). Furthermore, the neural network's learning rate was influenced by the type of transfer function used and the top model chosen acquired Tanh function at transfer function hidden and sigmoid for the transfer function output.

**Figure 3:**
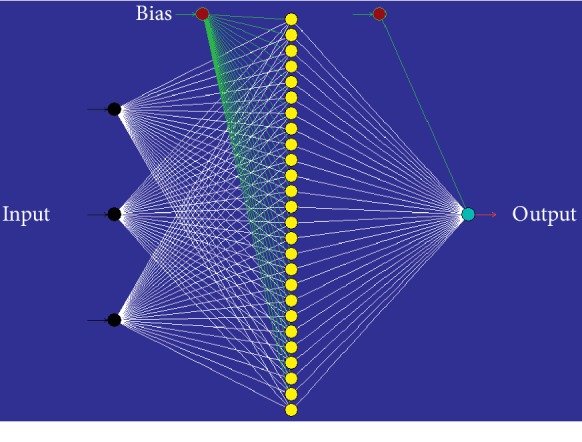
Neural network topology. The topology of multilayer normal feedforward neural network for the estimation of DB71 dye decolorization.

The finest topology for the prediction of DB71 dye decolorization consisted of 3-26-1 topology (Figure 3), and Table 4 shows that the best ANN model work was network number 1 containing 26 nodes of optimization and Tanh transfer function, and required a multilayer normal feedforward (MNFF) batch backpropagation (BBP) network. Both training and testing datasets possessed *R*^2^ of 0.999, which presented reduced error as opposed to other networks.

### 3.5. Determination of Optimum Point by Using RSM and ANN

The optimum level of various parameters obtained from RSM optimization was DB71 dye concentration of 150 ppm, yeast extract of 3 g/L, and pH of 6.645 with an overall decolorization of 92.2% as measured at 587 nm (Table 5). To validate this, the experiments were performed based on the predicted optimum condition to compare the experimental outcomes with the predicted outcomes. The average triplicate's experimental value of decolorization was 86.13% compared to the predicted value of 92.2% decolorization with 6.58% deviation. A previous study on DB71 dye decolorization by solar degradation shows that greater dye concentration inhibits the dye decolorization and only about 70% decolorization was obtained at 100 ppm [74] and only 71% of DB71 color removal by *P. fluorescens* D41 was obtained in the presence of glucose [75]. In comparison, the mix bacterial culture was able to decolorize DB71 dye better than the conventional method and single bacterial isolate due to the catabolic agreement between the microorganisms.

**Figure 4:**
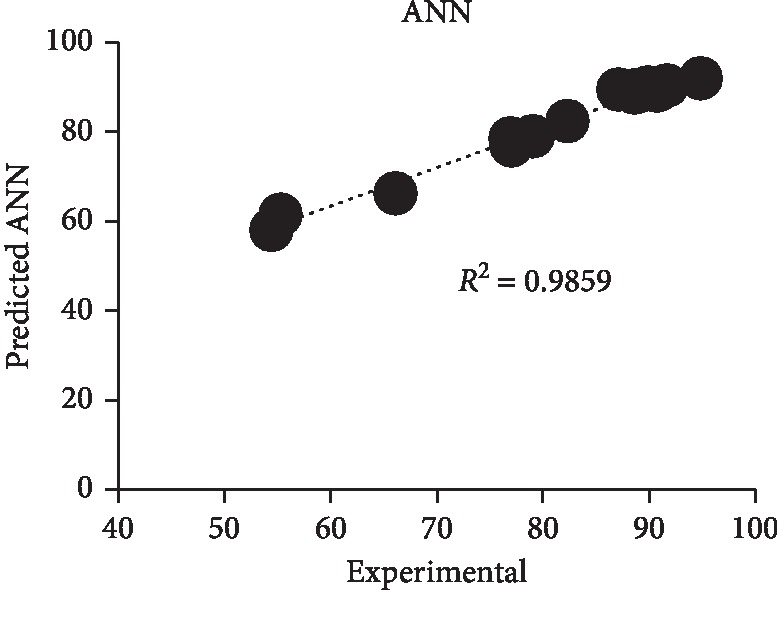
ANN predicted versus actual experimental data values for DB71 dye decolorization.

The optimum level of various parameters obtained from ANN optimization was DB71 dye concentration of 150 ppm, yeast extract of 3 g/L, and pH of 6.645 with an overall decolorization of 89.9%. For validation of optimum points using ANN, the average triplicate's experimental value of decolorization was 86.5% as compared to the predicted value of 89.9% of decolorization with a deviation smaller than RSM at around 3.78%. The ANN model could accurately fit with the experimental data as the experimental value and ANN predicted value show a close relationship. Incredibly good nonlinear fitting effects were obtained because the model predicted values were very close to the actual values (Figure 4). *R*^2^ was close to 1.0 at 0.9859 and this shows that ANN gave a good prediction.

### 3.6. Three-Dimensional Analysis

**Figure 5:**
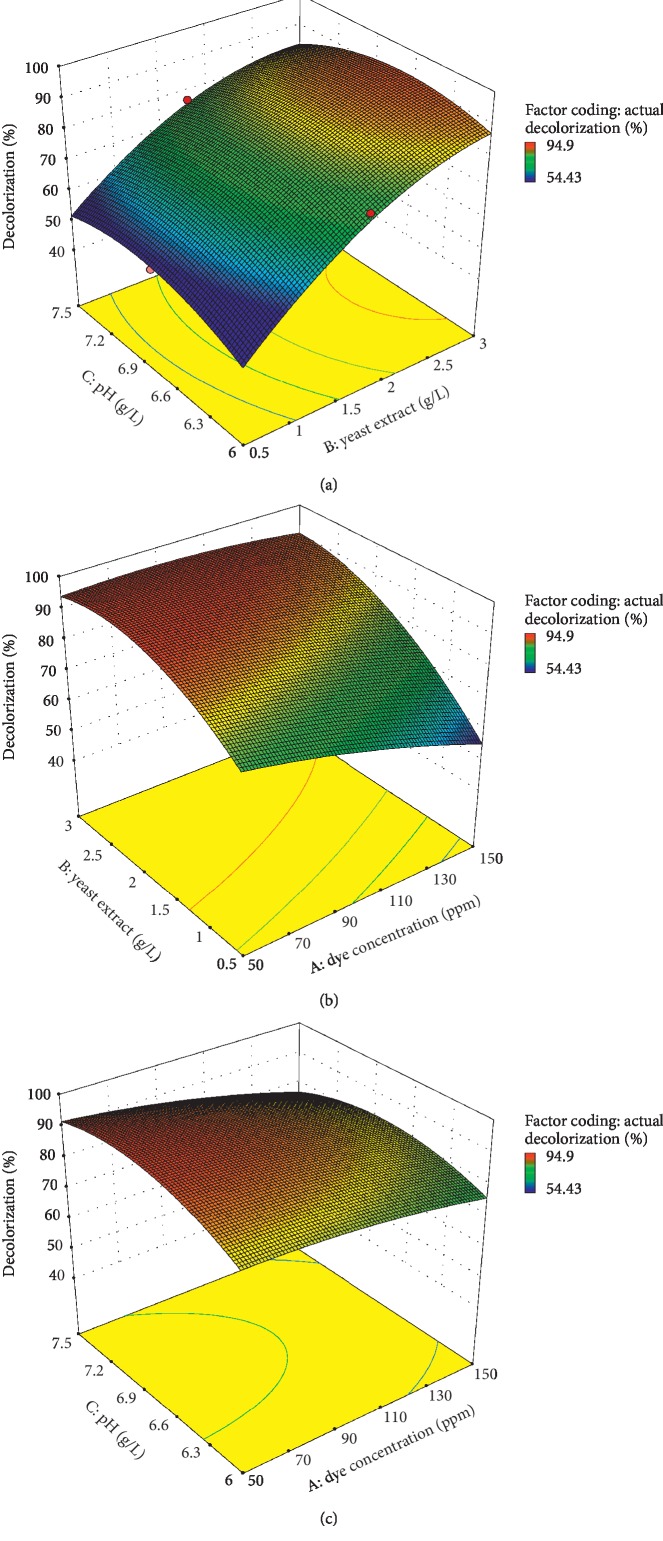
Three-dimensional plots showing the effect of (a) pH and yeast extract, (b)yeast extract and dye concentration, and (c)pH and dye concentration and their mutual effect on the decolorization of Direct Blue 71 dye. Other variables are constant: pH (6.645), yeast extract (3 g/L), and dye concentration (150 ppm).

By keeping the value of another variable constant, the impact of two variables at once was evaluated via RSM 3D surface plot. The contours depicted the optimal value of the variable that reveals the utmost DB71 dye decolorization (response). The effect of yeast extract and pH on the percent of decolorization (Figure 5(a)) was highlighted through the 3D response surface. The result displayed that as the yeast extract increased, the decolorization of dye also increased. The pH showed that decolorization increased as pH increased until the optimum condition was obtained. Both surface plots demonstrated the optimum concentration of yeast extract and pH were obtained at 3 g/L and pH 6.645, respectively. Yeast extract was identified as an effective enhancer for promoting higher decolorization performance. The regeneration of NADH during the reduction of azo dyes using microorganisms releases the electron donors and yeast, which is regarded as the organic nitrogen sources being a vital media supplement in the process [76]. As shown in Table 3, factor B (yeast extract) was a significant parameter since its *p* value was about 0.0001, which was much lower than 0.05.

The correlated effect of dye concentration and pH to dye decolorization signified that as the dye concentration increased, decolorization percent was likewise increased, and as pH increased, the decolorization increased as well before it reached the optimum point (Figure 5(b)). Both surface plots showed that the optimum concentrations of pH occurred at 6.645 with the dye concentration of 150 ppm. Any adjustments in medium pH greatly affected some biological functions, including enzymatic processes, element transport over the membrane, and the signaling pathways [77]. Neutral initial pH was favored by the majority of bacteria for the greatest growth, and KMK48 bacterium can degrade sulfonated azo dyes after only 36 hours in neutral pH [78]. Maximum decolorization process at higher pH has also been reported by halophilic bacteria out of genus *Halomonas* [79], and in contrast, Georgiou et al. [80] reported a slightly acidic pH of 6.6 for dye decolorization using acetate-consuming bacteria. Accordingly, bacterial cell metabolism and nutrients intakes were greatly affected by the surrounding medium pH.

The effect of dye concentration and yeast extract towards DB71 dye decolorization was displayed on a three-dimensional plot, in which as the dye concentration and yeast extract increased, the decolorization was also increased (Figure 5(c)). Both surface plots indicated that the optimum concentrations of dye concentration and yeast extract were gained at 150 ppm and 3 g/L, respectively. The elliptical plot obtained showed that there is a relationship concerning these two parameters. The increased initial dye concentration caused a steady rise in decolorization capacity. Dubin and Wright (1975) reported a lack of any effect on decolorization rate due to different dye concentrations. This observation was agreeable with a nonenzymatic decolorization process that was regulated by processes which were independent of the dye concentration [81].

**Figure 6:**
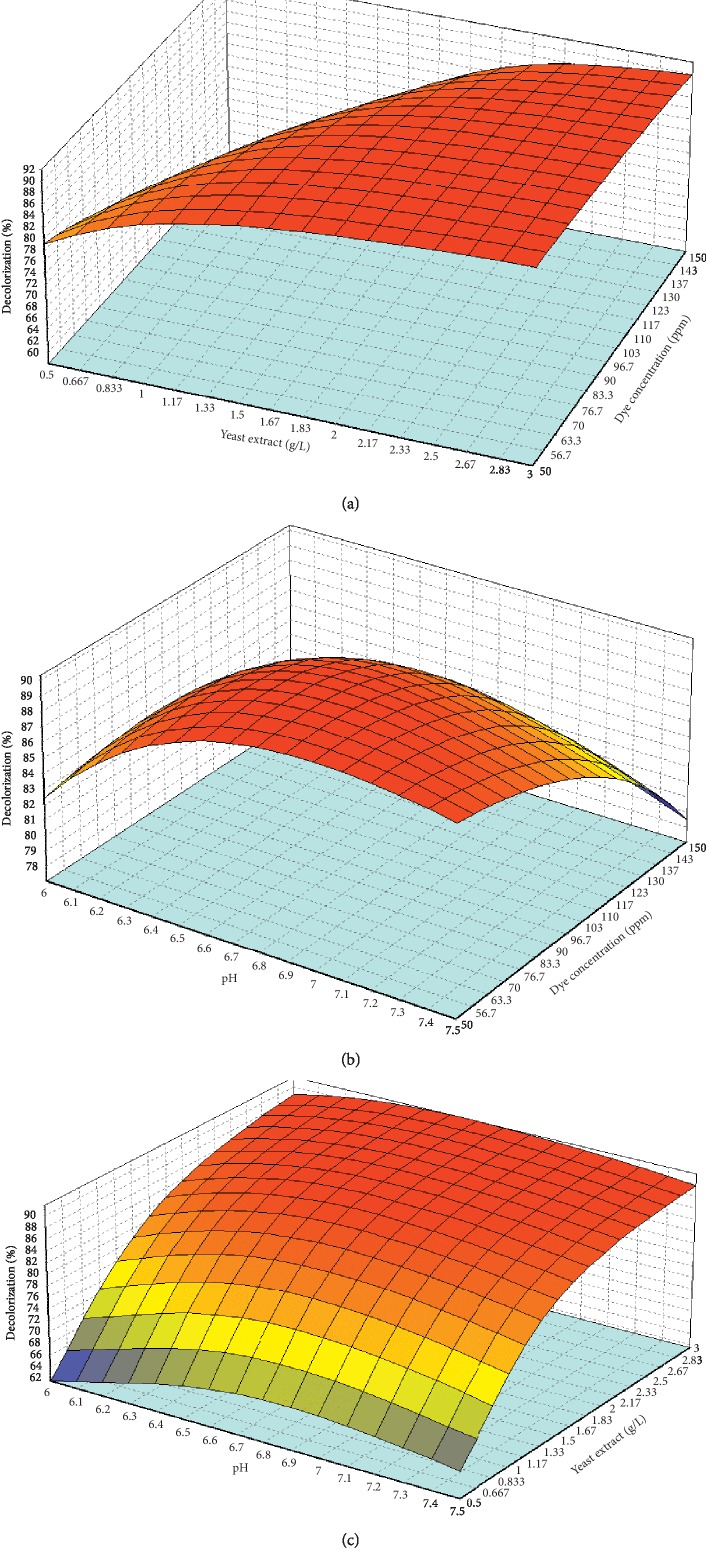
AAN response surface for (a) dye concentration versus yeast extract, (b) dye concentration versus pH, and (c) yeast extract versus pH with dye decolorization as a response.

The way that the factors of dye concentration, yeast extract, and pH corresponded with the DB71 dye decolorization was presented on 3D response surface plots by ANN (Figures 6(a)–6(c)). The effect of dye concentration and yeast extract on the decolorization of dye is displayed in Figure 6(a). Gradual increase of decolorization % was observed as the dye concentration increased and yeast extract (g/L) increased until it reached the optimal point. The study by Chang and Lin [82] also signified that an adequate supply of yeast extract is critical for the *Pseudomonas luteola* strain to achieve stability in the fed‐batch decolorization processes. The 3D plot from Figure 6(b) shows the effect of pH and concentration of dye on dye decolorization. Based on the result, given that dye concentration and pH increased, the decolorization percent increased as well until it achieved the optimum point. Higher pH causes the decrement in decolorization and maximum decolorization was observed at pH 6.7. A similar result has been reported before by Kapdan [83] that used mixed bacterial consortium to decolorize textile dye and higher dye concentration inhibits the microbial decolorization. Next, Figure 6(c) shows that the decolorization % increased as yeast extract increased and pH increased until it reached the optimum point for the highest dye decolorization. Yu et al. [84] reported a significant effect of pH on dye decolorization because of the highest activity of *Pseudomonas* sp. The strain was observed at a pH range of 7–8, and 50% decrement in activity was observed when the pH was varied.

### 3.7. Residual Analysis (Error Analysis)

The dependability and accuracy of RSM and ANN models were evaluated through a relative study [85] by using *R*^2^ to analyze the model's precision ability [54]. A good model ought to have *R*^2^ close to 1.0.  Table 6 shows that ANN displayed a larger value of *R*^2^ (0.990) compared to RSM (0.980), but the efficiency of the regression model does not only depend on *R*^2^ because additional evaluation factors such as AAD were highly recommended to validate several models [86]. A small value of AAD, which is close to zero, shows a less chance of error in prediction and was displayed as a good model. ANN model (0.04) possesses a smaller value of AAD compared to the RSM model (0.045) as shown in Table 6. Thus, the critically higher predictive and accuracy potential of the ANN model compared to the RSM model was obtained based on the relative *R*^2^ and AAD values.

## 4. Conclusions

The mixed bacterial culture shows an amazing ability to decolorize Direct Blue 71 dye's triazo bond without the presence of carbon and nitrogen sources in anaerobic condition, rendering it more applicable for in situ application. A successful optimization was shown by using RSM and ANN techniques. 86.13% and 86.5% of validated decolorization were obtained in optimized condition predicted by RSM and ANN subsequently at a concentration of 150 ppm. Furthermore, a relative study on RSM and ANN could cover several cons of each technique as well as emphasize the error in the experimental data. Hence, a higher value of *R*^2^ (0.99) and a lower value of AAD (0.04) show that the ANN model holds a better prediction for optimization of the dye decolorization compared to the RSM model.

## Figures and Tables

**Table 1:** Lower limit and upper limit of Box–Behnken Design.

Parameters	Unit	Lower limit	Upper limit
Dye concentration	ppm	50	150
Yeast extract	g/L	0.5	3
pH		6	7.5

**Table 2:** Box–Behnken matrix for experimental design and predicted response using RSM and ANN.

Run	A: dye conc (ppm)	B: yeast extract (g/L)	C: pH (g/L)	Decolorization (%)	Predicted RSM (%)	Predicted ANN (%)
1	100	1.75	6.75	90.97	89.84	89.356
2	100	1.75	6.75	87.15	89.84	89.382
**3**	**50**	**3**	**6.75**	**94.9**	94.1	**92.076**
4	150	3	6.75	89.86	92.09	89.862
5	150	1.75	6	77.03	74.87	77.03
6	**150**	**0.5**	**6.75**	**54.43**	55.23	**58.118**
7	100	3	6	88.69	88.62	88.689
8	100	0.5	7.5	66.18	66.25	66.18
**9**	**150**	**1.75**	**7.5**	**77.01**	76.14	**78.499**
10	100	1.75	6.75	88.57	89.84	89.347
11	100	3	7.5	89.8	88.44	89.799
12	50	0.5	6.75	79.06	76.83	79.06
**13**	**100**	**0.5**	**6**	**55.31**	56.67	**61.547**
14	50	1.75	6	82.38	83.25	82.381
15	100	1.75	6.75	90.72	89.84	89.328
**16**	**100**	**1.75**	**6.75**	**91.77**	89.84	**90.356**
17	50	1.75	7.5	89.22	91.38	89.222

ANN training dataset—unbold numbers. ANN testing dataset—bold numbers.

**Table 3:** Analysis of variance (ANOVA) for the fitted quadratic polynomial model for optimization of Direct Blue 71 dye decolorization.

Source	Sum of squares	df	Mean square	*F* value	*p* value prob > *F*	
Model	2448.23	9	272.03	47.15	<0.0001	Significant
A: dye conc	278.83	1	278.83	48.33	0.0002	
B: yeast extract	1465.3	1	1465.3	253.96	<0.0001	
C: pH	44.18	1	44.18	7.66	0.0278	
AB	95.94	1	95.94	16.63	0.0047	
AC	11.76	1	11.76	2.04	0.1964	
BC	23.81	1	23.81	4.13	0.0817	
A^2^	15.67	1	15.67	2.72	0.1433	
B^2^	293.16	1	293.16	50.81	0.0002	
C^2^	177.72	1	177.72	30.8	0.0009	
Residual	40.39	7	5.77			
Lack of fit	25.76	3	8.59	2.35	0.2138	Not significant
Pure error	14.63	4	3.66			
Cor total	2488.62	16				
					*R * ^2^	0.9838
					Adjusted *R*^2^	0.9629

**Table 4:** The effect of different neural network architecture and topologies on *R*^2^ and AAD in the estimation of DB71 dye decolorization obtained in the training and testing of neural networks.

Network	Model	Learning algorithm	Connection type	Transfer function output	Transfer function hidden	Training set *R*^2^	DC	Testing set *R*^2^	DC
**1**	**4.26.1**	**BBP**	**MNFF**	**Sigmoid**	**Tanh**	**0.999**	**0.980**	**0.999**	**0.950**
2	4.26.1	BBP	MNFF	Tanh	Tanh	0.990	0.980	0.980	0.870
3	4.26.1	BBP	MNFF	Tanh	Sigmoid	0.991	0.982	0.900	0.790
4	4.26.1	BBP	MNFF	Sigmoid	Linear	0.98	0.97	0.99	0.900

**Table 5:** Predicted and experimental value for the responses at optimum condition using response surface methodology (RSM) and an artificial neural network (ANN).

Model	A: dye conc (ppm)	B: yeast extract (g/L)	C: pH	RSM/ANN predicted (decolorization)	Experimental validation	Deviation
RSM	150	3.0	6.645	92.2	86.13	6.58
ANN	150	2.90	6.7	89.90	86.50	3.78

**Table 6:** Absolute deviation, *R*^2^, adjusted *R*^2^, and AAD of RSM and ANN models.

Residual analysis	RSM	ANN
*R * ^2^	0.980	0.990
Adjusted *R*^2^	0.978	0.989
Absolute average deviation (AAD)	0.045	0.040

## Data Availability

The response surface methodology (RSM) and artificial neural network (ANN) data used to support the findings of this study are available from the corresponding author upon request.

## Nomenclature

### Abbreviations

DB71: Direct Blue 71
RSM: Response surface methodology
ANN: Artificial neural network
AAD: Absolute average deviation
PDW: Printing and dyeing wastewater
MSM: Minimal salt medium
DNA: Deoxyribonucleic acid
ANOVA: Analysis of variance
GPS: Global positioning system
MNFF: Multilayer normal feedforward
BBP: Batch backpropagation
RMSE: Root mean square error

### Symbols

3D: Three-dimensional
NADH: Electron carrier
*R *^2^: Coefficient of determination
rpm: Revolutions per minute
*yi *_exp_: Experimental responses
*yi *_cal_: Measured responses
*p*: Number of experimental runs
*n*: Number of the experimental data
% w/v: Percentage weight per volume
*p* value: Probability value
Tanh: Hyperbolic tangent.
